# Asymptomatic Giant Right Ventricular Myxoma in an Adolescent: A Rare Case Report With Favorable Surgical Outcome

**DOI:** 10.1002/jcu.70260

**Published:** 2026-05-07

**Authors:** Aizezi Maihemu, Jin Bai, Chongjun Qi, Jian Liu

**Affiliations:** ^1^ Department of Cardiac Surgery The Third People's Hospital of Xinjiang Uygur Autonomous Region Urumqi Xinjiang China

**Keywords:** echocardiography, primary cardiac tumors, right ventricular myxoma, right ventricular outflow tract, surgery

## Abstract

Right ventricular (RV) myxomas account for < 5% of cardiac myxomas and are often asymptomatic, easily overlooked. We report a 13‐year‐old female with a giant RV myxoma (70 × 35 × 49 mm) extending to the main pulmonary trunk, detected via routine adolescent health screening. Multimodal imaging (TTE, CT, MRI) facilitated diagnosis, and complete surgical resection was performed. Postoperative histopathology confirmed the diagnosis, with favorable short‐term outcomes. This case highlights that proactive TTE‐based screening, multimodal imaging evaluation, and early surgical intervention are crucial for improving the prognosis of patients with right ventricular myxoma. This case highlights that proactive TTE‐based screening, multimodal imaging evaluation, and early surgical intervention are crucial for improving the prognosis of patients with right ventricular myxoma.

## Graphical abstract

We report a 13‐year‐old female with a giant right ventricular myxoma extending to the pulmonary trunk. The tumor was diagnosed by multimodal imaging and completely resected, with favorable short‐term outcomes.

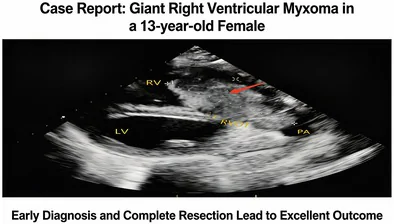

## 1 Introduction

Primary cardiac tumors are relatively rare, with an autopsy incidence of 0.001%–0.28% (Bussani et al. 2020). Among them, cardiac myxoma is one of the dominant subtypes—a systematic review and meta‐analysis involving 8849 patients (Rahouma et al. 2020) showed that 5140 cases (61.6%) out of 8346 primary cardiac tumors were cardiac myxomas. A study by Mittle et al. (2005) demonstrated that approximately 75% of cardiac myxomas are located in the left atrium, while a small minority occur in the right ventricle (RV), accounting for < 5%. This disease lacks specific clinical manifestations; even in the asymptomatic state, it may cause severe complications such as embolism (systemic embolism from left‐sided tumors and pulmonary embolism from right‐sided tumors), intracardiac obstruction, congestive cardiac failure (CHF), and cardiac arrhythmia. Surgical resection is the first‐line treatment (Tyebally et al. 2020; Okongwu and Olaofe 2025). This article reports an extremely rare case of a giant RV myxoma (extending to the main pulmonary trunk) in a 13‐year‐old female patient, aiming to provide a reference for the diagnosis and treatment of such rare and complex cases.

## 2 Case Report

A 13‐year‐old female patient was advised to undergo transthoracic echocardiography (TTE) after a systolic ejection murmur was auscultated at the 3rd and 4th intercostal spaces on the left sternum during a physical examination. The TTE revealed a mass in the RV, and the patient was therefore referred to our hospital. She reported occasional chest tightness and shortness of breath on exertion for approximately 1 month. Her family history was negative for congenital heart disease, cardiomyopathy, sudden cardiac death, and thrombotic tendency. On admission, her blood pressure was 110/75 mmHg, and body temperature, pulse, and respiratory rate were all within the normal range. Physical examination identified a systolic ejection murmur at the 3rd and 4th intercostal spaces on the left sternal border, as well as significant jugular vein distension. The level of N‐terminal pro‐B‐type natriuretic peptide (NT‐proBNP) was elevated (1934 pg/mL, reference range 0–100 pg/mL), with no other abnormalities detected.

**FIGURE 1:**
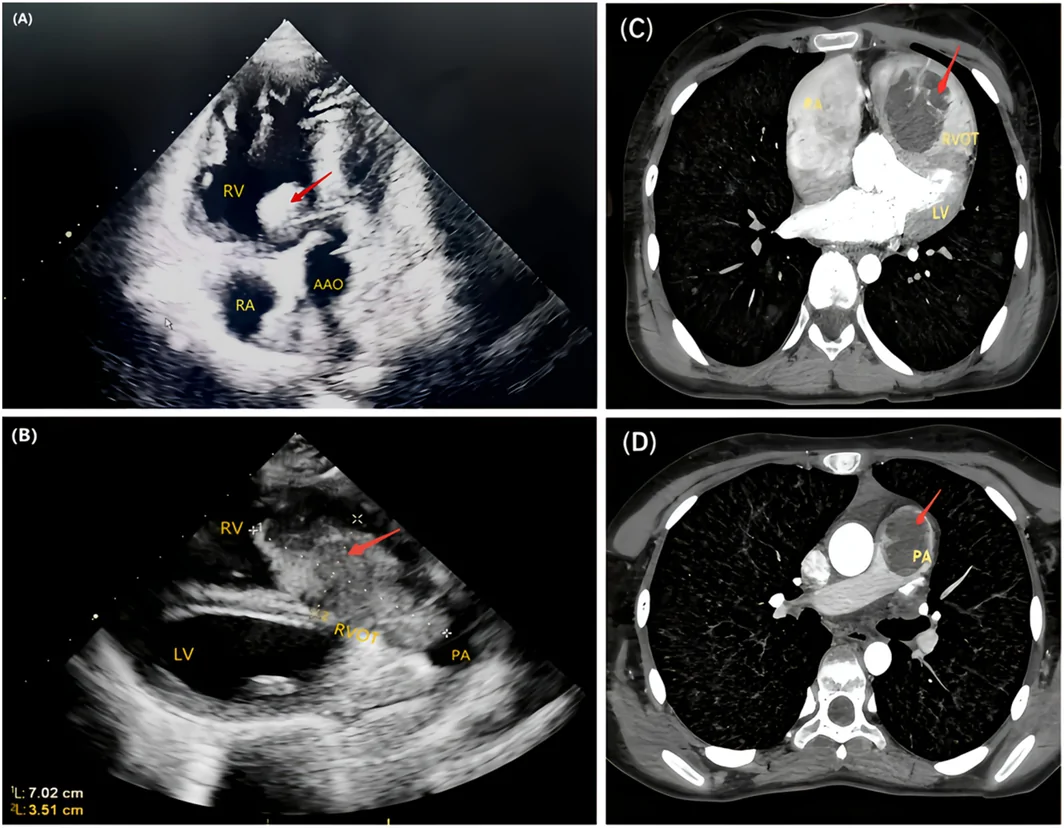
Preoperative imaging findings. (A–B) Transthoracic echocardiography images: (A) A mass in the RV (arrow). (B) The mass extending into the RVOT and PA (arrow). (C–D) Computed tomography images: (C) A mass in the RVOT (arrow). (D) A mass in the PA (arrow). AAO, ascending aorta; LV, left ventricle; PA, pulmonary artery; RA, right atrium; RV, right ventricle; RVOT, right ventricular outflow tract.

**FIGURE 2:**
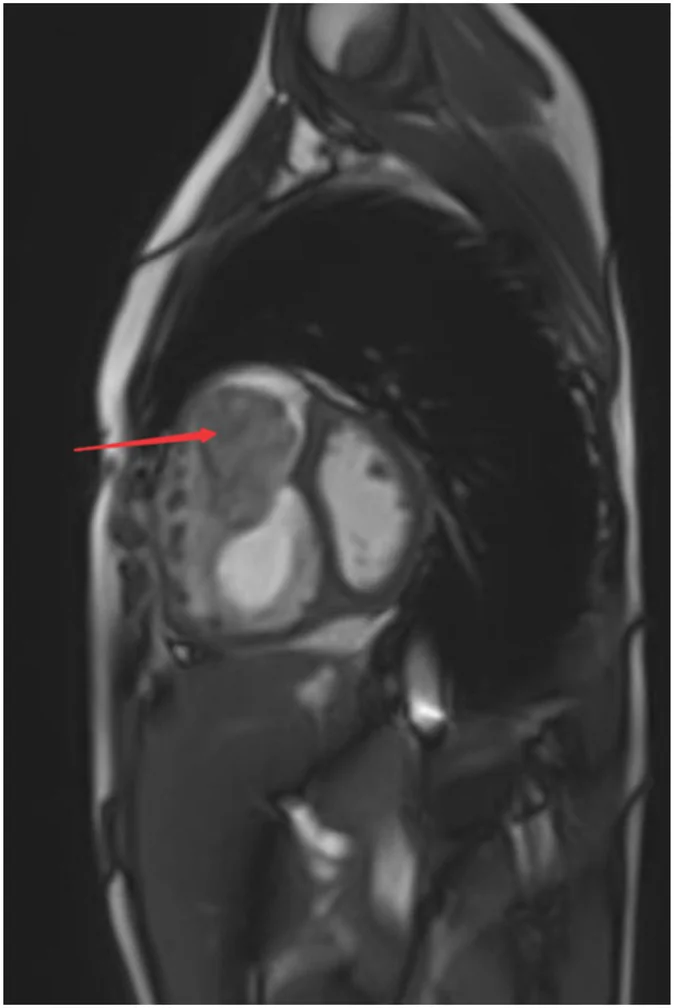
Sagittal view of cardiac magnetic resonance imaging showing RV mass (arrow).

No abnormalities were found on chest X‐ray or electrocardiogram (ECG). However, TTE showed an irregularly shaped soft tissue mass (70 × 35 × 49 mm) attached to the RV wall. During systole, the mass protruded into the right ventricular outflow tract (RVOT) and proximal pulmonary artery (PA). Dilatation of the RV and right atrium (RA) was observed, accompanied by mild tricuspid regurgitation, RVOT stenosis, and an ejection fraction of 59% (Figure 1A,B). Chest computed tomography (CT) confirmed a low‐density heterogeneous mass in the RV with flocculent enhancement, which extended through the RVOT to the base of the main pulmonary trunk (Figure 1C,D). To clarify the nature of the lesion, further cardiac magnetic resonance imaging (MRI) was performed, which suggested a neoplastic lesion. The high signal intensity on T2‐weighted imaging indicated the presence of myxoid components in the lesion (Figure 2). Combining clinical and imaging findings, cardiac myxoma was highly suspected. Given that the patient had a giant RV myxoma protruding into the RVOT and proximal PA, which posed a severe risk of sudden cardiac death due to complications such as outflow tract obstruction and embolism, urgent surgery was planned.

**FIGURE 3:**
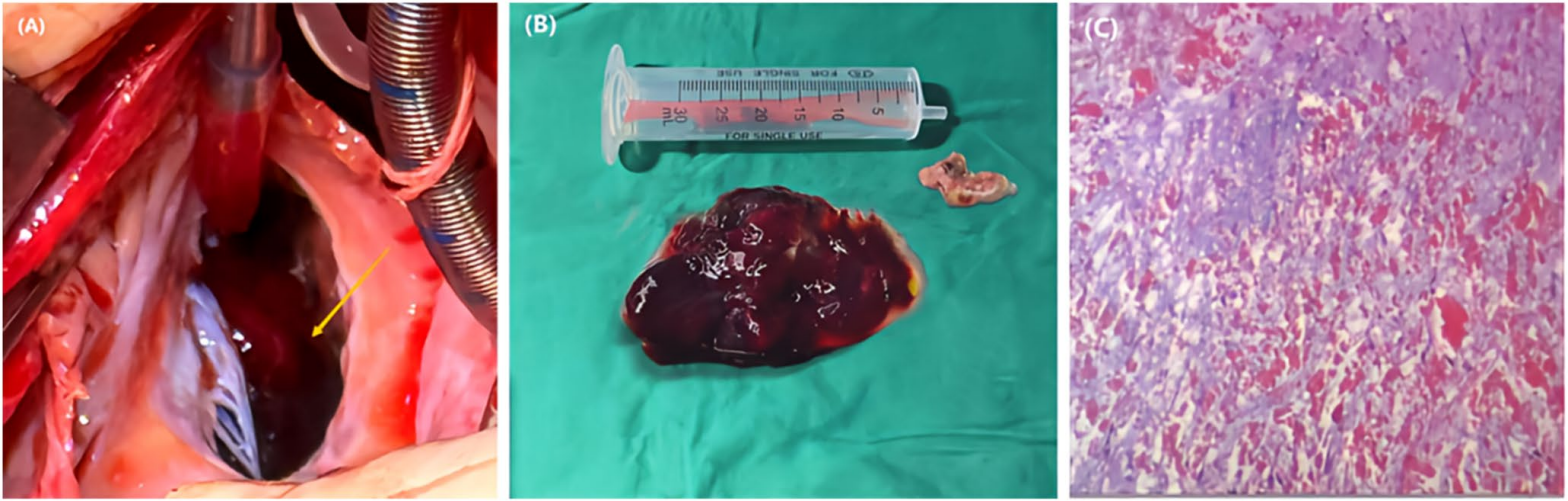
Intraoperative findings and histopathological examination of the cardiac myxoma: (A) Exposed RV tumor; (B) Completely resected intact tumor; (C) Irregular myxomatous cells sparsely dispersed in myxoid stroma.

Myxoma resection was performed via median sternotomy. Cardiopulmonary bypass was established using an aortic cannula and a double‐lumen venous cannula under moderate hypothermia, and the heart was arrested with cold blood cardioplegic solution. After incising the RA, the myxoma was visualized through the atrial incision, with its stalk attached to the anterior wall of the RV, and was completely resected (Figure 3A,B). Histopathological examination showed that the total size of the tumor tissue was 70 × 35 mm, mainly composed of myxoid components (Figure 3C). Postoperative re‐examination by TTE confirmed complete resection of the myxoma, with mild improvement in tricuspid regurgitation and recovery of RV function. The patient had an uneventful postoperative course and was discharged on the 10th day. No complications or disease recurrence were observed on TTE at the 1‐month postoperative follow‐up.

## 3 Discussion

Myxoma is the most common benign cardiac tumor after papillary fibroelastoma, typically presenting as a mobile mass attached to the endocardial surface via a stalk (Maleszewski et al. 2022). It mostly originates from the fossa ovalis (Dujardin et al. 2000), with 75% of cases occurring in the LA and 15%–20% in the RA. Rare cases of myxomas originating from the RV have been reported (Mittle et al. 2005)—a retrospective study by Cetin et al. (2010) on 9756 consecutive open‐heart surgery cases at a single center between 1985 and 2007 showed that RV myxomas accounted for an extremely low proportion of only 4.2% (1 out of 24 myxoma cases). Despite its extreme clinical rarity, it remains an important component of cardiac oncology practice, and timely diagnosis and standardized treatment are crucial for improving patient prognosis (Tyebally et al. 2020; Okongwu and Olaofe 2025). Although the tumor is histopathologically benign with a favorable prognosis, its hazards extend far beyond the tumor itself—due to individual variations in tumor size and intracardiac location, it is prone to causing significant hemodynamic disturbances, which in turn induce severe complications including intracardiac obstruction, systemic, and pulmonary embolism, as well as systemic symptoms mimicking connective tissue diseases and inflammatory diseases.

The myxoma reported in this case is extremely rare in terms of both location and size. The lesion is located in the RV and is giant in size (70 × 35 × 49 mm). Although current methods for monitoring the growth rate of myxomas are not yet well‐established, existing studies (Rubio Alvarez et al. 2013; Stowe et al. 2023) have confirmed that myxomas grow relatively rapidly. More notably, myxomas often lack overt clinical manifestations in the early stage; even with the development of a giant tumor, the patient in this case only complained of occasional chest tightness and dyspnea on exertion in the late stage without early discomfort, which renders early detection extremely challenging. Such giant RV myxomas with the features of rapid growth and early asymptomatic status carry a high risk of inducing severe complications such as embolism, intracardiac obstruction, and congestive heart failure (CHF), and may even lead to sudden cardiac death (Li et al. 2022). This clinical characteristic further highlights the necessity of regular cardiac screening for adolescents, especially for the early identification of asymptomatic cardiac lesions. Xinjiang has long carried out mobile medical service campaigns in remote and impoverished areas, providing free screening for congenital heart disease and other heart‐related diseases. This patient was found to have a heart murmur during a physical examination in this campaign, and further TTE indicated a RV tumor, successfully avoiding such a tragic outcome. Murai and Nakashima (2025) also emphasized the importance of regular examinations in adolescents for detecting asymptomatic heart diseases.

Notably, this case further confirms that TTE serves as an indispensable first‐line modality for the clinical screening of cardiac diseases, with its application value being particularly prominent in asymptomatic populations. Featuring the technical advantages of non‐invasiveness, radiation freedom, cost‐effectiveness, and bedside implementability, TTE enables a comprehensive assessment of cardiac anatomical structures and cardiac function status, acting as a core examination method for identifying cardiac structural abnormalities in routine health screening. In this case, following the detection of a systolic ejection murmur during the physical examination, TTE first identified the giant space‐occupying lesion in the RV, serving as the primary diagnostic basis for clinical lesion localization and laying a foundation for subsequent multimodal imaging evaluation and surgical planning.

In addition to the above clinical characteristics, a striking discordance was observed in this patient regarding laboratory indicators and clinical symptoms. A markedly elevated NT‐proBNP (1934 pg/mL) was detected in the absence of overt clinical symptoms of heart failure or severe hemodynamic compromise. This finding highlights the superiority of objective laboratory and imaging screening over sole reliance on clinical symptoms: the elevation of NT‐proBNP reflected subclinical myocardial stretch and mild RV dysfunction caused by the compression of the RV and RVOT by the giant myxoma, even though the patient had not yet developed overt CHF or persistent dyspnea. Such subclinical hemodynamic disturbances are difficult to identify without objective screening, which further justifies the necessity of combining routine physical examination with objective screening tools such as TTE and laboratory tests.

Although TTE plays an irreplaceable role in the early screening and initial diagnosis of cardiac lesions, it has inherent limitations in the detailed evaluation of complex cardiac anatomical structures. Outflow tract obstruction caused by RV myxomas is clinically rare, and the curved triangular anatomical structure of the RV makes a single imaging technique insufficient for comprehensive diagnosis. In this specific case, TTE successfully realized the initial localization of the RV giant mass and identified its dynamic protrusion into the RVOT and proximal PA during the cardiac cycle, but it failed to accurately delineate the tumor's exact three‐dimensional spatial extent, the precise anatomical relationship between the tumor and the main pulmonary trunk, and the internal density characteristics of the lesion—these limitations increased the difficulty of formulating a detailed surgical resection plan. To address these limitations and optimize surgical planning, we adopted a multimodal imaging combined evaluation strategy before surgery: on the basis of TTE's initial diagnosis, chest enhanced CT clearly displayed the low‐density heterogeneous nature of the RV mass and its flocculent enhancement pattern, and further clarified the spatial adjacency between the lesion and intrathoracic blood vessels (including the RVOT and the base of the main pulmonary trunk); cardiac MRI further accurately assessed ventricular wall motion and identified the high signal intensity on T2‐weighted imaging, which confirmed the presence of myxoid components in the lesion and ruled out additional myocardial or valvular abnormalities.

This multimodal imaging approach achieved detailed multi‐dimensional visualization of the lesion's attachment site, size, and boundaries, providing a precise anatomical and pathological basis for the formulation of surgical resection plans and effectively avoiding the risk of incomplete tumor resection. Surgery is the key to successful treatment of myxomas—a systematic review and meta‐analysis (Oktaviono et al. 2024) showed that patients with cardiac myxomas have excellent postoperative outcomes, with an early mortality rate of only 1.27%, a late mortality rate of 4.7 per 1000 person‐years, and a recurrence rate of 0.5 per 1000 person‐years. Therefore, once a diagnosis of cardiac myxoma is confirmed, timely tumor resection is required. It should be noted that vigorous palpation and other cardiac manipulations should be avoided before surgery to reduce tumor stimulation; for high‐risk patients (with large, pedunculated, and highly mobile tumors), strict activity restriction is necessary when appropriate to avoid acute risks; and gentle manipulation during surgery is required to prevent embolism, while achieving complete tumor resection to reduce recurrence and long‐term complications.

## 4 Conclusion

In summary, the clinical diagnosis of right ventricular myxomas is highly challenging due to its low incidence and insidious symptoms. However, early diagnosis and surgical intervention are crucial for reducing the risk of complications such as CHF, intracardiac obstruction, embolism, and even sudden cardiac death. Therefore, it is essential to enhance clinical awareness of this disease: on the one hand, targeted screening should be strengthened for patients without clear predisposing factors, the ability to identify atypical cases should be improved, and regular health check‐ups should be advocated to achieve early detection; on the other hand, the application of TTE‐centered multimodal imaging in cardiac evaluation should be emphasized, and surgical resection should be performed as early as possible after definitive diagnosis, so as to ultimately reduce the risk of mortality in patients.

## Funding

The authors have nothing to report.

## Ethics Statement

The studies involving human participants were approved by the Ethics Committee of the Third People's Hospital of Xinjiang Uygur Autonomous Region (Approval No.: XJSQ2025121713). Prior to the publication of this case report and all accompanying images, written informed consent was obtained from the patient in accordance with the ethical standards of the Declaration of Helsinki and relevant institutional regulations.

## Conflicts of Interest

The authors declare no conflicts of interest.

## Data Availability

The raw data supporting the conclusions of this article will be made available by the authors, without undue reservation.
